# Measurement of Coupling Coordination Degree and Spatio-Temporal Characteristics of the Social Economy and Ecological Environment in the Chengdu–Chongqing Urban Agglomeration under High-Quality Development

**DOI:** 10.3390/ijerph182111629

**Published:** 2021-11-05

**Authors:** Jiangjun Wan, Yuxin Li, Chunchi Ma, Tian Jiang, Yi Su, Lingqing Zhang, Xueqian Song, Haiying Sun, Ziming Wang, Yutong Zhao, Kaili Zhang, Jinxiu Yang

**Affiliations:** 1 School of Architecture and Urban-Rural Planning, Sichuan Agricultural University, Chengdu 611130, China; wanjiangjun@sicau.edu.cn (J.W.); liyuxin@stu.sicau.edu.cn (Y.L.); 2019325016@stu.sicau.edu.cn (T.J.); 41360@sicau.edu.cn (L.Z.); shy370923@outlook.com (H.S.); knightzm@outlook.com (Z.W.); 201608383@stu.sicau.edu.cn (Y.Z.); zhangkaili_5223248@163.com (K.Z.); 2 State Key Laboratory of Geohazard Prevention and Geoenvironment Protection, Chengdu University of Technology, Chengdu 610059, China; machunchi17@cdut.edu.cn; 3 Rural Development Research Institute, Sichuan Academy of Social Sciences, Chengdu 610041, China; esribaihc@gmail.com; 4 School of Management, Chengdu University of Information Technology, Chengdu 610225, China; sxq@cuit.edu.cn; 5 College of Economics, Sichuan Agricultural University, Chengdu 611130, China

**Keywords:** Chengdu–Chongqing urban agglomeration, social economy, ecological environment, coupling coordination degree

## Abstract

With rapid urbanization and industrialization, ecological disorders and environmental degradation have become serious, and the promotion of the coordinated development of the social economy and ecological environment is not only a pressing problem to be solved, but also an important step towards sustainable development. The coordinated development of the social economy and eco-environment is conducive to sustainable development. Considering the Chengdu–Chongqing urban agglomeration as a case study, this paper adopts panel data and establishes an index system to evaluate the coupling coordination degree (CCD) between the social economy and ecological environment based on the concept of high-quality development. From the perspective of time and space, the changing laws and characteristics of the CCD are analyzed, and the key factors affecting it are determined using regression analysis. The results show the following: (1) the CCD between the social economy and ecological environment of the Chengdu–Chongqing urban agglomeration presents a low level overall; (2) the CCD in more developed regions is significantly higher than that in less developed regions; thus, the characteristics of spatial differences are obvious; (3) the urbanization rate, ratio of actual use of foreign capital and GDP, ratio of total export-import volume and GDP, proportion of days with good air quality, and per capita public green space area are the main factors affecting the coordinated development of the social economy and ecological environment in the Chengdu–Chongqing urban agglomeration; and (4) Chongqing has obvious endogeneity. Finally, corresponding policy recommendations are provided aimed at promoting rapid economic development in the Chengdu–Chongqing urban agglomeration while focusing on environmental protection and promoting high-quality economic development with ecological environmental protection, while putting forward decision-making suggestions for high-quality development of urban agglomerations.

## 1. Introduction

Since “Reform and Opening” up was introduced in 1978, the urbanization of China has grown at an unprecedented rate. In 2018, China’s urbanization rate reached 59.85%. Considering the current development trend, the urban population is expected to exceed 1 billion in the next 20 years [1]. The urbanization rate of Chengdu and Chongqing in 2018 was 73.12% and 65.5%, respectively. Urban agglomeration is the highest form of spatial organization in the mature stage of urban development, and it has a significant driving effect on regional economies. The imbalance of regional economic development in China urgently requires Western regions to play the leading role of core cities. Therefore, the construction of the Chengdu–Chongqing urban agglomeration has become an important national strategy for the development of Western China. The relationship between the economy and ecological environment has always been the focus in the development of urban agglomerations. The imbalance of the regional economic development in China urgently required Western regions to develop into an urban agglomeration that matched the order of the Eastern coastal urban agglomeration, giving rise to the Chengdu–Chongqing urban agglomeration. On the one hand, the ecological environment provides resource conditions for economic and social development and improves people’s living conditions. On the other hand, excessive and unsustainable development will lead to many environmental problems, such as water pollution, reduction of farmland, greenhouse effect, and scarcity of resources, while severe environmental problems will restrict the development of the social economy. The coordinated development between the above is the core of realizing regional sustainable development. In recent years, the Chinese government has paid increasingly more attention to the high-quality development of the economy and presented the concept of high-quality development. Among the five connotations, “green” means sustainability and the construction of ecological civilization, and “coordination” emphasize the urban and rural coordination, the regional coordination, and the coordination of the economic society. The “Chengdu–Chongqing Urban Agglomeration Development Plan” advocates integrating the concept of respecting nature and people-oriented green urbanization into the development and construction of urban agglomerations, implementing environmental governance, building green urban agglomerations, and promoting green and sustainable development. Therefore, under the current urgent and unbalanced development situation of urbanization and the concept of high-quality development, it is necessary to promote ecological co-construction and environmental co-governance. Furthermore, the research on the social economy and ecological environment of the Chengdu–Chongqing urban agglomeration is particularly important.

Currently, the research on high-quality development of urban agglomerations is an urgent and necessary focus, and existing research in this area is relatively weak. This paper considers 16 cities of the Chengdu–Chongqing urban agglomeration as a study case, using the coupling coordination degree (CCD) model and combining the five connotations of the concept of high-quality development to evaluate the coordination relationship between the social economy and ecological environment from the perspective of urban agglomerations. In order to achieve the coordinated development of the social economy and ecological environment, and promote the sustainable development of urban agglomerations, certain suggestions are provided.

## 2. Literature Review

The current research on the relationship between the economy and ecological environment is relatively complete. The environmental Kuznets curve (EKC) reveals the inverted U-shaped relationship between the economy and ecological environment [2]. It points out that when the level of economic development is low, the degree of environmental pollution is lighter and gradually deteriorates with economic growth; when the economic development reaches a certain level, that is, after reaching a certain critical point or “turning point,” with a further increase in per capita income, the degree of environmental pollution gradually slows down, and the environmental quality gradually improves. Many scholars have confirmed this hypothesis through empirical research. Lee and Oh [3] used China’s prefecture-level panel data from 2003 to 2010 and the fixed effects model to confirm that there is an inverted U-shaped relationship as well as an N-shaped relationship between China’s income and environmental pollution. Zhao et al. [4] studied the relationship between urbanization and “ecologicalization” in the Yangtze River Delta through the improved EKC model, which confirmed the EKC hypothesis and further found that the level of urbanization of economically developed areas is higher than that of underdeveloped areas at the turning point of the curve. However, the EKC curve also has many limitations. Essentially, the relationship between the environment and economy may be more complicated than the EKC assumption. Kijima et al. [5] confirmed that there is a non-linear relationship between urbanization and the environment, but this curve does not conform to the inverted U-shaped relationship of EKC. As the trend of urbanization sweeps the world, many empirical studies also focus on analyzing the relationship between urbanization and the ecological environment or a single environmental element. Fan et al. [6] explored the coupling relationship between urbanization and air quality in Shandong Province from 2014 to 2017 and found that highly coordinated cities often have more developed economies and higher levels of urbanization, for instance, the coastal areas of Shandong. Yang et al. [7] analyzed the relationship between the geo-ecological environment and urbanization in Chongqing from 1990 to 2015 and found that the two systems conformed to the inverted U-shaped law. Li et al. [8] used the panel data of Lianyungang from 2000 to 2008 and constructed a comprehensive indicator system of urbanization level from four aspects: population urbanization, economic urbanization, social urbanization, and spatial urbanization, using the pressure-state-response (PSR) environmental assessment method. Through empirical analysis, it was concluded that social urbanization and environmental control have the greatest impact on CCD between urbanization and the environment. The CCD models are generally used to solve these problems. Coupling is a physical concept that refers to the degree of interaction and mutual influence between two or more systems or elements. Coupling coordination is used to represent the non-linear interaction between systems. Many scholars use the CCD to evaluate the coordinated development relationship between two or more systems. Shi et al. [9] used a CCD model with geography and time-weighted regression (GTWR) to study the coupling coordination and spatio-temporal heterogeneity of economic development and ecological environment in 17 tropical and subtropical regions in China from 2003 to 2016. The results reveal that economic development and the ecological environment are in an intermediate coupling coordination stage, and there is significant temporal and spatial heterogeneity. Zhang and Li [10] developed an improved CCD model based on a genetic algorithm and found that there is a U-shaped relationship between China’s urbanization and natural disasters, and the CCD also changed from imbalance to a transitional stage. Liu et al. [11] also used the above methods to analyze the CCD between Nansi Lake catchment’s social economy and the water environment. Chen et al. [12] studied the CCD of China’s carbon emissions and the ecological environment from 2009 to 2015 based on the CCD model and found that the two barely reached a dynamic balance. Based on remote sensing data, Ariken et al. [13] analyzed the relationship between urbanization and eco-environment in China’s typical arid area of Yanqin Basin using the CCD model.

Scholars have also researched urban agglomerations from the perspective of the eco-environment. The Beijing–Tianjin–Hebei urban agglomeration has always been the focus of researchers as it is located in the capital economic circle. Ariken et al. [14] combined ecosystem services and ecosystem health to assess the ecological risks of the Beijing–Tianjin–Hebei urban agglomeration, and found that the ratio of artificial surface should be controlled below 70%, which undertook a comprehensive evaluation of the impact of land use planning on the ecosystem. Wang et al. [15] studied the factors affecting the coordinated development of urbanization and ecological environment of the Beijing–Tianjin–Hebei urban agglomeration, while Li et al. [16] studied the impact of urban expansion on atmospheric humidity, exploring that the effect of urban expansion on humidity was the strongest in winter, which give scientific support for urban planning. Zeng et al. [17] used a scenario-based optimization framework to study the relationship between the population, economy, resources, and the environment, and proposed a comprehensive strategy that can improve it. The research on the Beijing–Tianjin–Hebei urban agglomeration is relatively abundant. Saha et al. [18] used remote sensing data to analyze the impact of land use on surface temperature in the process of urbanization in several urban agglomerations in India and found that the surface temperature and urban heat island effect of urban areas are higher. Luo et al. [19] studied the decoupling relationship between economic growth and resource environment in the Central Plains urban agglomerations in China, which is helpful for regional planning.

China presented the concept of high-quality development in 2017, indicating that China’s economy has shifted from rapid growth to a stage of high-quality development. Since then, many experts and scholars began research on the background of high-quality development, and have also carried out the construction of high-quality development evaluation index systems. Ma et al. [20] analyzed the high-quality development of China’s regional economy in different dimensions, and the results show that the high-quality development of China’s economy presents a non-equilibrium characteristic of decreasing regionally from the East to the Central regions and then to the West. Chen et al. [21] used high-quality development and cities along the Yellow River Basin as the research objects, while also using the obstacle degree model to analyze the obstacles. They discussed the impact of the obstacles on the urban ecological level and proposed corresponding policy suggestions. Chen et al. [22] analyzed the factors affecting the ecological carrying capacity of high-quality development in 286 cities in China and found that most of the cities with high ecological carrying capacities were provincial capitals.

Judging from existing research, particularly in developing countries such as China, the current research on the economy and ecological environment has become more comprehensive and abundant due to the rapid development of urbanization and the economy. However, most of them focus on a certain city or an administrative region. As such, there is a lack of research on urban agglomerations. Most of the research on the concept of high-quality development only focuses on economics and lacks in-depth discussion in combination with the ecological environment. In 1990, China’s “Urban Planning Law” stipulated to “strictly control the scale of large cities,” otherwise the Matthew effect would result in “urban diseases.” The formation of urban agglomerations is a natural reflection of economic development and industrial layout, and also the main form and successful model of urbanization in developed countries. Therefore, based on the connotation of high-quality development, this article discusses the coupling coordination relationship between the social economy and ecological environment from the perspective of urban agglomerations, and provides a certain theoretical reference for the construction of ecological urban agglomerations.

## 3. Methods and Data Source

### 3.1. Study Area

**Figure 1:**
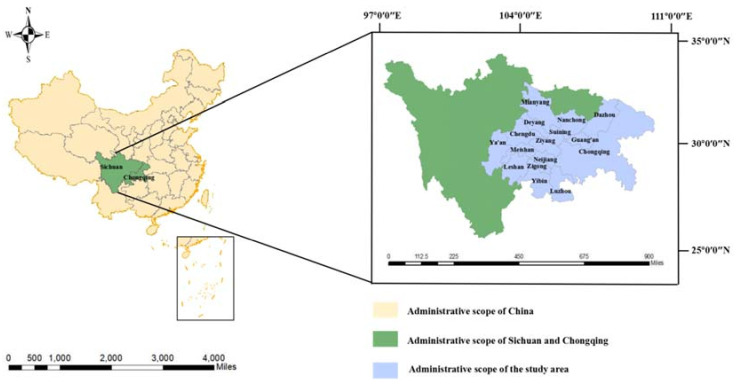
Location of the study area.

**Figure 2:**
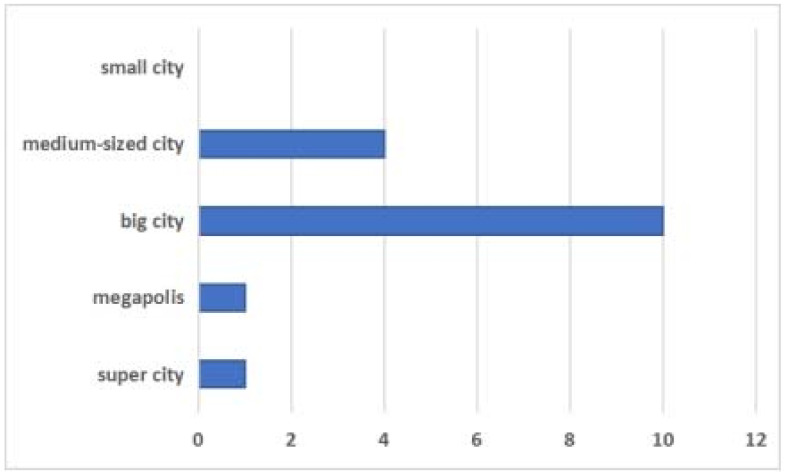
City scale distribution map of the Chengdu–Chongqing urban agglomeration.

The Chengdu–Chongqing urban agglomeration is located in southwestern China, which is centered between Chengdu and Chongqing. The detailed location of the research area is shown in Figure 1, and the city scale distribution map is shown in Figure 2. According to the new city scale classification standard (urban resident population), cities are divided into five types, they are small city (under 500,000), medium-sized city (from 500,000 to 1 million), big city (from 1 million to 5 million), megapolis (from 5 million to 10 million), and super city (more than 10 million), respectively. It is the largest urban agglomeration area in the Western region. It is also an important platform for the development of the Western region and the strategic support of the Yangtze River Economic Belt. The Chengdu–Chongqing urban agglomeration has a total area of 185,000 km² with complex topography, which is high in the West and low in the East. It is generally dominated by hills, plains, and basins, and is one of the five major urban agglomerations in China, the others being the Yangtze River Delta, the Pearl River Delta, the Beijing–Tianjin–Hebei agglomeration, and the middle reaches of the Yangtze River. Currently, the urbanization of the Chengdu–Chongqing urban agglomeration is still at a relatively low level, without an obvious city structure and appropriate city hierarchical structure, which is the lack of the small city and super city, presenting the phenomenon of “big in the middle and small at the ends”. According to existing research, Chengdu and Chongqing have outstanding high-quality economic and social development, with obvious fault characteristics and obvious regional differences in ecological environment quality. As an important urbanization region in China, the Chengdu–Chongqing urban agglomeration has obvious geographical advantages, connecting the East to the West and the North to the South. Further, it is highly developed economically, has an increasingly perfect urban system, and has close economic, social, and cultural links. The two leading cities, Chengdu and Chongqing, are not far apart and have advantages in terms of integration over other urban agglomerations. To cultivate and develop the Chongqing urban agglomeration, to give full play to its unique advantages of connecting the southwest and northwest, and domestic and foreign countries, and to promote the interaction between the “One Belt And One Road” and the Yangtze River Economic Belt strategy, it is conducive to accelerate the development of the Central and Western regions, expanding new spaces for national economic growth, ensuring national security, and optimizing the layout of the country. Based on the promotion of the above-mentioned policies, the Chengdu–Chongqing urban agglomeration is booming.

### 3.2. Data Source

Based on the “Chengdu–Chongqing Urban Agglomeration Development Plan” issued by the National Development and Reform Commission and the Ministry of Housing and Urban–Rural Development, this paper takes 14 prefecture-level cities in Sichuan Province, Chengdu, the capital of Sichuan Province, and Chongqing, the municipality directly under the central government, as the research objects. The research period is set from 2009 to 2018. Considering that this paper focuses on the development of the whole urban agglomeration led by Chengdu and Chongqing, as well as the size of Chongqing and the lack of data, all districts and counties in Chongqing are combined and treated as an evaluation unit. Considering the desirability and scientific aspects of the original data, in this paper, the ecological environment and social-economic statistics are derived from the China City Statistical Yearbook, Sichuan Statistical Yearbook, Chongqing Statistical Yearbook, and the statistical bulletins of various cities and government work reports. The missing values are predicted and supplemented by linear regression. Quantitative analysis, spatial analysis, and spatial visualization processing of the data are undertaken with the help of SPSS, ArcGIS, Stata, and other software.

### 3.3. Research Framework

**Figure 3:**
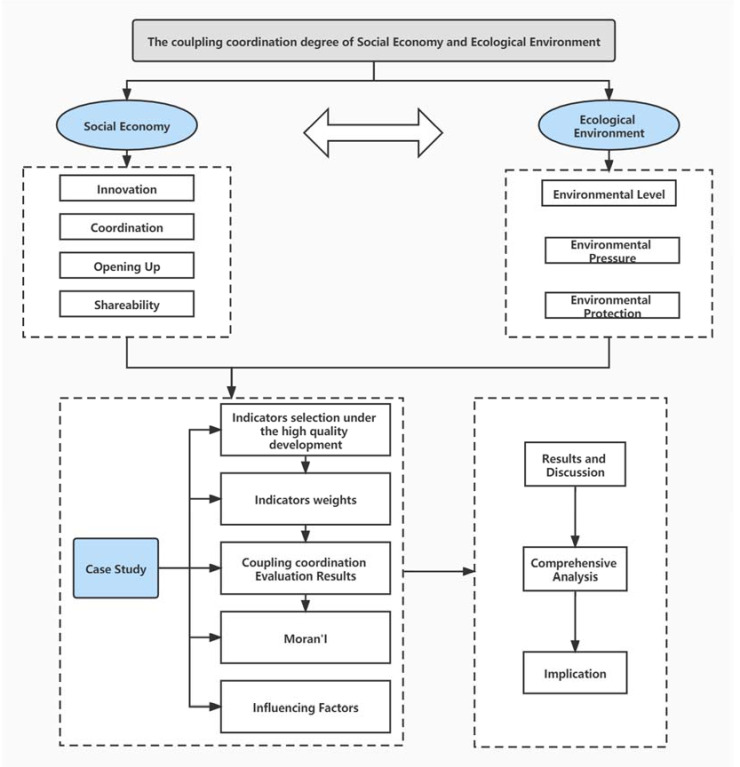
The framework of measurement of coupling coordination degree and spatio-temporal characteristics of the social economy and ecological environment.

In order to research on the coupling coordination relationship of social economy and ecological environment, this study determined the indexes under the concept of high-quality development, and then calculated the index weights. Based on SPSS 21.0 statistical analysis software, we made a comprehensive analysis to measure the coordination degree and the coupling coordination degree of social economy and ecological environment. Then, the Moran index was calculated based on ArcGIS 10.4 software and analysis the CCD characteristics from the perspective of time and space. The factors influencing CCD were determined by Stata15.1. Finally, we present a comprehensive analysis and discussion in this paper according to the results. The specific implementation steps are as follows (Figure 3).

### 3.4. Evaluation of CCD of the Social Economy and Ecological Environment under the High-Quality Concept

(1) Construction of the indicator system

Based on the principles of being systematic, integral, scientific, and operational, this paper combines the five concepts of innovation, coordination, openness, sharing, and green of the high-quality development concept to establish the two sub-systems of the social economy and ecological environment. As shown in Table 1, the ecological environmental system is decomposed into three dimensions of ecological environment pressure, eco-environment level, and ecological environment protection based on the PSR environmental quality assessment model, with a total of eight indicators. The social economy system includes four dimensions: innovation, coordination, openness, and sharing, with a total of 11 indicators.

Due to the different properties of each evaluation index, it is necessary to standardize the data to eliminate the influence of dimension. In this paper, the commonly used min-max standardization is adopted, which is a linear normalization method, and all the original data are processed to 0–1 value.

If there are r years, n cities, and m indexes, the ositive indicator is as follows:

(1) $$y_{\theta ij}=\frac{x_{\theta ij}-\min(x_{\theta ij})}{\max(x_{\theta ij})-\min(x_{\theta ij})}$$

and the negative indicator is:

(2) $$y_{\theta ij}=\frac{\max(x_{\theta ij})-x_{\theta ij}}{\max(x_{\theta ij})-\min(x_{\theta ij})}$$

where, $x_{\theta ij}$ represents the original and normalized values of the j-th index in the i-th city in the θ-th year, and $\max(x_{\theta ij})$ and $\min(x_{\theta ij})$ represent the maximum and minimum values, respectively.

(2) CCD model

In this paper, the CCD model is adopted to explore the coupling coordination level between the social economy and ecological environment. First, the improved entropy method is used to determine the index weight. As panel data are used in this paper to realize the comparison between different years, this paper uses Yang Li’s method [23] for reference, which improves the method of entropy, and time variables are added to make the analysis results more comprehensive and reasonable. The evaluation index of the two systems of the social-economic system and ecological environment is then calculated. Finally, the coupling degree and CCD are calculated.

Step 1. Calculate the information entropy of each index:

(3) $$e_{j}=-k\sum_{\theta }\sum_{i}(p_{\theta ij}\ln y_{\theta ij})$$

where, $p_{\theta ij}=\frac{y_{\theta ij}}{\sum_{\theta }\sum_{i}y_{\theta ij}}$, *k* > 0, and *k* = 1/ln(rn).

Step 2. Determine the index weight:

(4) $$w_{j}=\frac{d_{j}}{\sum_{j}d_{j}}$$

where, $d_{j}=1-e_{j}$.

Step 3. Calculate the evaluation value of the two systems:

(5) $$s_{1}=\sum_{j=1}^{n}(w_{j}y_{\theta ij})$$

(6) $$s_{2}=\sum_{j=1}^{n}(w_{j}y_{\theta ij})$$

where, $S_{1}$ represents the economy development, and ${}_{S_{2}}$ represents the eco-environment system.

Step 4. Coupling degree:

(7) $$C=\sqrt{\frac{S_{1}•S_{2}}{(S_{1}+S_{2})/2}}$$

Step 5. Coupling coordination degree:

(8) $$D=\sqrt{C•T}$$

where, representing the comprehensive development, C is the coupling degree between $S_{1}$ and $S_{2}$, α,β are undetermined coefficients, and $\alpha \in \beta \in \left[0,1\right]$ and α+β=1 indicate the contribution coefficients of $S_{1}$ and $S_{2}$, respectively. We assume that α,β is equally important of the two systems, so the values of α,β can be set as 0.5 D, representing the coupling coordination degree between the two systems. The coupling degree reflects the intensity of mutual influence between the two systems, and the coupling coordination degree model can further reflect if the development of the system is coordinated to some extent.

(3) Spatial autocorrelation

In order to further explore the spatial relationship of CCD, we use Moran’s I to conduct spatial autocorrelation analysis. Moran’s I index takes values in the range [−1,1]. A positive value of the Moran’s I indicates that the variables are positively autocorrelated on the spatial units, and the closer the value to 1, the closer the relationship between the spatial units. That is, high-value spatial units are adjacent to high-value spatial units and low-value spatial units are adjacent to low-value spatial units. If Moran’s I index is 0, there is no spatial relationship between the cells, i.e., high-value and low-value spatial cells are completely randomly distributed. Further, a negative value of Moran’s I indicates that the variables are negatively autocorrelated on spatial units, and the closer its value is to −1, the greater the difference between spatial units and the more dispersed the distribution, i.e., high-value spatial units are adjacent to low-value spatial units. Local Moran’s I test specifically reflects the degree of local spatial agglomeration in each region, including “high-high”, “low-low”, “low-high”, “high-low” agglomeration patterns and “no significant spatial correlation”, and the LISA significance test reflects its spatial distribution pattern and significance degree.

Global Moran’s *I*:

(9) $$I=\frac{n\sum_{i=1}^{n}\sum_{j=1}^{n}w_{ij}(x_{i}-\bar{x})(x_{j}-\bar{x})}{\sum_{i=1}^{n}\sum_{j=1}^{n}w_{ij}\sum_{i=1}^{n}{\left(x_{i}-\bar{x}\right)}^{2}}=\frac{\sum_{i=1}^{n}\sum_{j=1}^{n}w_{ij}(x_{i}-\bar{x})(x_{j}-\bar{x})}{S^{2}\sum_{i=1}^{n}\sum_{j=1}^{n}w_{ij}}$$

Local Moran’s *I*:

(10) $$I=\frac{\left(x_{i}-\bar{x}\right)}{s^{2}}\sum_{j=1}^{n}w_{ij}(x_{j}-\bar{x})$$

Among them, $S^{2}=\frac{1}{n}\sum_{i=1}^{n}(x_{i}-\bar{x}{)}^{2}$, $\bar{x}=\frac{1}{n}\sum_{i=1}^{n}x_{i}$. $x_{i}$ denotes the observation of the *i*th region, $S^{2}$ is the sample variance, n is the total number of regions, and $w_{ij}$ is the (*i*,*j*) element of the spatial weight matrix.

(4) Regression model

In order to explore the degree of influence and significance of various indicators on the CCD, the CCD is considered as an explained variable and 19 indicators are considered as explanatory variables to conduct a regression analysis of panel data from 2009 to 2018. Firstly, the data are assumed to have individual effects, and the xtreg command is used for regression. The xtreg command performs simple clustering and difference on the data, which is more suitable for the processing of panel data [24]. Whether the fixed effect model or random effect model is used is determined by the Hausman test. All these operations are undertaken using Stata 15.1.

## 4. Results

### 4.1. Spatio-Temporal Characteristics of the Social Economy and Ecological Environment Associated with the CCD

#### 4.1.1. Temporal Characteristics of the CCD

Based on the above CCD model, the CCD of the two systems, namely, social economy and ecological environment, was evaluated from 2009 to 2018 for 16 cities in the Chengdu–Chongqing urban agglomeration, as shown in Table 2. It was found that the overall level of the CCD between the social economy and ecological environment in the Chengdu–Chongqing urban agglomeration is relatively low. In the past decade, the CCD of each city increased. The overall CCD increased from 0.10 to 0.30 in 2009 to 0.14 to 0.40 in 2018. The CCDs of Chongqing, Chengdu, Deyang, and Mianyang have been at the forefront and ranked firmly in the top four. In 2009, the CCD of Chengdu was the highest at 0.30, and in 2018, Chongqing had the highest CCD at 0.40. As shown in Table 3, Chongqing has the largest variation of the CCD, and the smallest variation was witnessed in Zigong and Nanchong at only 0.01. The ranking of Nanchong dropped from 6th in 2009 to 12th in 2018, with the largest decline in ranking; the ranking of Luzhou rose by five, from 10th to 5th, Ya’an rose three places, and the ranking of other cities did not change significantly. Chongqing, Chengdu, Deyang, and Mianyang are ranked in the top four, both in terms of change in the CCD and ranking. The rankings of Neijiang and Dazhou did not change, where they were ranked 10th and 16th in 2009 and 2018, respectively.

According to previous studies, the CCD can be classified according to the level, as shown in Table 4. Certainly, most cities have not shown coordination in terms of social economy and ecological environment in the past decade. Since 2009, the CCDs of various cities has been gradually increasing with Chongqing, Chengdu, Mianyang, and Deyang at the forefront of growth, while the growth rate of other cities is relatively low.

From the above analysis, it can be observed that the CCD of social economy and ecological environment in the Chengdu–Chongqing urban agglomeration is increasing, and the trend is gradually reaching basic coordination, but most of the CCDs of cities still present no coordination in the development process.

#### 4.1.2. Spatial Characteristics of the CCD of Social Economy and Ecological Environment

**Figure 4:**
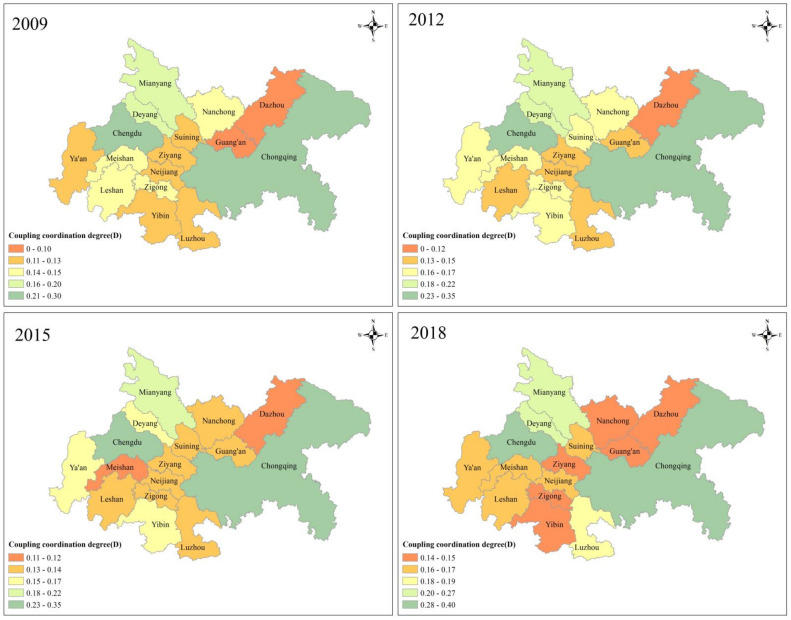
The spatial distributions of the CCD in the years 2009, 2012, 2015, and 2018.

Based on the ArcGIS10.4 software, the spatial distributions of the CCD of the research area in 2009, 2012, 2015, and 2018 are depicted in Figure 4.

The spatial characteristics of the CCD of the Chengdu–Chongqing urban agglomeration in China are as follows. First, the CCD of most of the cities has barely changed and has not substantially improved in the past 10 years. As of 2018, only Chongqing has nearly reached basic coordination, while Chengdu, Deyang, and Mianyang are also gradually approaching basic coordination. Other cities have not reached basic coordination, and there remains a certain distance to it. Only Deyang rose from no coordination in 2009 to low coordination in 2018. Second, Chengdu and Chongqing, the two central cities, not only have an obvious dual-core dominance in social-economic development, but also have a much higher degree of CCD when compared to other cities. Therefore, the regions with a higher CCD between social economy and ecological environment are mainly the more developed cities. Third, Chengdu and Chongqing did not promote the CCD of other cities. The CCD of these cities has not been positively affected by the high-quality development of Chengdu and Chongqing, particularly Ziyang, the city in the middle of Chongqing and Chengdu. Fourth, the CCD of the Chengdu–Deyang–Mianyang economic belt is at the forefront of the Chengdu–Chongqing urban agglomeration, indicating that the integrated development of the Chengdu–Deyang–Mianyang area is excellent. Finally, none of the cities have reached a state of basic coordination, but Chongqing and Chengdu currently show a trend to achieve it.

### 4.2. Spatial Autocorrelation Analysis

The coupling coordination degrees of 2009, 2012, 2015, and 2018 were selected as the observed values to conduct spatial autocorrelation analysis in GeoDa software, and the Queen neighborhood was adopted to set the spatial weight matrix.

#### 4.2.1. Global Moran’s I Test

The global Moran’s I generated by global autocorrelation is shown in Table 5. It can be seen that the CCD of the social economy and ecological environment in Chengdu–Chongqing urban agglomeration is always less than zero, presenting a significant negative correlation during the research period; that is, with the discretization of spatial distribution, the correlation becomes significant, and this relationship has a trend of gradual decline but the last 3 years were more dynamic. It increased from −0.190 in 2009 to −0.110 in 2018, indicating that the spatial difference of the CCD between the social economy and ecological environment is getting bigger and bigger.

#### 4.2.2. Local Moran’s I Test

**Figure 5:**
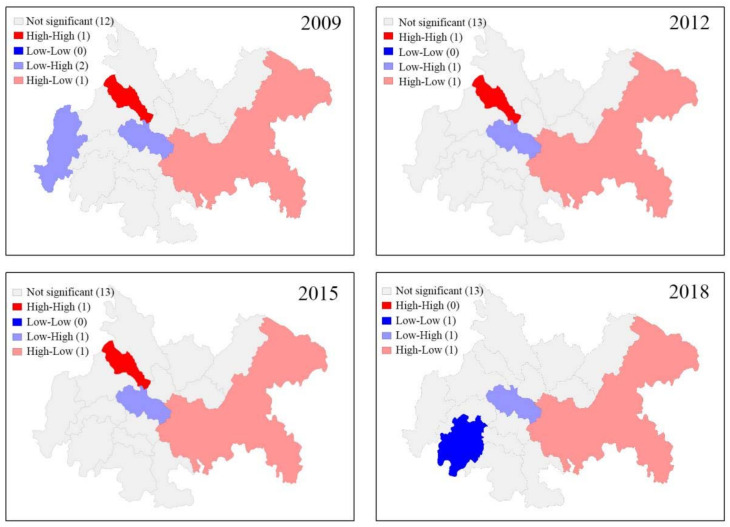
The LISA agglomeration map of 2009, 2012, 2015, and 2018.

From Figure 5, we can know that Chongqing has shown the “high-low” agglomeration pattern since 2009. Deyang showed the “high-high” agglomeration pattern in 2009, 2012, and 2015, which turned into the “no significant spatial correlation” pattern in 2018. Ziyang has always shown the “low-high” agglomeration pattern during the research period. Leshan had shown the “no significant spatial correlation” pattern since 2009 and turned into the “low-low” agglomeration pattern in 2018. Ya’an showed the “low-high” agglomeration pattern in 2009 and has shown the “no significant spatial correlation” pattern since 2012.

### 4.3. Factors Influencing CCD

This study used the fixed effects regression model to explore the factors that affect the CCD between the social economy and ecological environment of the Chengdu–Chongqing urban agglomeration. In terms of the goodness-of-fit of the model, the closer the coefficient of determination (R-squared) of the model sample is to 1, the better the fit of the model. The estimated result of this model is 0.961, indicating that the difference between the estimated value of the model and the actual value is very small, and the overall effect of the model is feasible. Concerning previous experience, it is assumed that it is significant at the 1% confidence level, that is, the *p*-value is less than 0.01. The results show that the urbanization rate, ratio of actual use of foreign capital and GDP, ratio of total export-import volume and GDP, proportion of days with good air quality, and per capita public green space area have significant impacts on the CCD of the social economy and ecological environment (Table 6). It passed the 10% significance test, which shows that these variables have a significant correlation with CCD. The proportion of education expenditure in GDP and sewage treatment rate passed the 5% significance test. There is no significant correlation with other variables. The regression coefficient shows that the proportion of days with good air has the greatest impact on the CCD, where the proportion of days with good air quality has increased by 1 unit, and the level of coupling and coordination increased by 0.07 units.

## 5. Discussion

Based on the above results, the phenomenon of dual-core dominance is obvious in the Chengdu–Chongqing urban agglomeration, especially in Chongqing, which is obviously an endogenous city and has not played a leading role in the surrounding cities. Ziyang has obvious positive effects and spillover to the surrounding cities. Chengdu and Chongqing not only have a high level of social-economic development, but are also ranked high in terms of the CCD, which is consistent with the research of Fan, Wang, Liu, and Liu [6]. This is the development cliff induced by the siphoning effect of big cities [25], where core cities, central cities, big cities, and cities with a dominant position can attract resource elements from surrounding cities, small- and medium-sized cities, and small towns. With the accumulation of resources, the attractiveness of central cities will become stronger and talents in surrounding cities will gradually drain, which creates the siphoning effect and has other negative effects, such as the poverty belt around Beijing and Tianjin. This requires core cities to play a leading role in providing equal development rights to small- and medium-sized cities, guide characteristic development of small- and medium-sized cities, reduce the excessive concentration of functions of large cities, and promote the spillover effect of large cities, while radiating and driving the development of small- and medium-sized cities. Except for Mianyang and Deyang, other cities in the Chengdu–Chongqing urban agglomeration are not greatly affected by the radiation of the two central cities, and the coupling coordinated development level of the social economy and ecological environment is very low. This could because the Chengdu–Deyang–Mianyang area is the region with the best development foundation in Sichuan, with obvious advantages in industrial development, the highest technological innovation, the highest level of urbanization, and the highest per capita GDP. These three cities not only have good economic foundations, but also have high levels of urban planning and environmental management. They also have close relationships and huge development potential. Other cities in the Chengdu–Chongqing urban agglomeration have a poor development foundation. Many cities, such as Leshan and Ya’an, have several chemical plants. If they are not strictly controlled, they will not drive the economy and will pollute the environment, which will have several negative impacts. From the perspective of resource development, the reason for the long duration of the serious imbalance of social economy and ecological environment in these cities may be that development and industries consumed more resources than expected. As such, it is challenging to realize coupling coordinated development [26].

Through regression analysis, it was identified that urbanization rate, ratio of actual use of foreign capital and GDP, ratio of total export-import volume and GDP, the proportion of days with good air quality, and per capita public green space area are notable factors. The result of urbanization rate is consistent with the research of Ariken et al. [12], which points out that the positive feedback effects of urbanization on the ecological environment are mainly distributed in southwest China and Shaanxi Province, while the negative effects are more concentrated in northwest China. In the Chengdu–Chongqing urban agglomeration, the urbanization rate reflects that the urban economy can be improved in the process of urbanization [27]; thus, the quality of the ecological environment, the overall development level of the region, and the industrial structure can be improved. In addition, to meet the demands of the increasing urban population, the government will improve infrastructure and public services, which can contribute to the coupling coordinated development of social economy and ecological environment to a certain extent. The National New-Type Urbanization Plan proposed to optimize the spatial layout and form of urbanization with urban agglomerations as the main body, and proposed that China build a total of 19 urban agglomerations during the 13th Five-Year Plan period. The comprehensive development of urban agglomerations is closely related to urbanization. The two factors of proportion of days with good air and per capita park green area are obvious and consistent with our assumption. City managers should strictly control air quality, build urban ecological parks, and optimize urban greening to improve the urban ecological level. For example, Chengdu implemented the “Ecological Zone around the City Plan”, and many ecological parks have been built in recent years, such as Nibatuo Park, Luxi River Ecological Zone, and Tianfu Park. In recent years, the Sichuan government has fully implemented the prevention and control of ozone pollution. The Department of Ecology and Environment has carried out assistance and guidance for ozone pollution stations in 10 key cities across the province in 2020, provided technical solutions, and effectively reversed the unfavorable situation of declining air quality. The government should pay attention to the economic structure when developing the economy. The proportion of foreign direct investment (FDI) in GDP represents the introduction of green production technology and advanced machinery and equipment through foreign investment, which can improve China’s level of productivity and improve the environmental pollution problem in China. Scholars have found that the policy environment of the country of origin and not the policy environment of the host country determines the positive or negative impact of FDI on the environment of the host country [28]. Therefore, the government should strictly control the local foreign-invested enterprises and raise relevant standards and requirements.

As an important platform for the development of the Western region, the Chengdu–Chongqing urban agglomeration must protect the ecological environment, while developing the economy to achieve high-quality development. Therefore, this paper can provide some reference for future development planning of the Chengdu–Chongqing urban agglomeration. Inevitably, this paper also has some limitations. The selection of indicators may not be comprehensive and perfect. Future research can further screen the indicators in combination with the current situation of regional development. The nine districts (the main urban area) of Chongqing play a vital role in economic development of Chongqing. With the main urban area of Chongqing as the main body, it radiates to other districts and counties, promoting the simultaneous development of 38 districts and counties of Chongqing. The total area of the main urban area of Chongqing accounted for about 6.6%, but the residents accounted for about 28%, and in 2020, the GDP exceeded 900 billion, accounting for about 39.5%. It can be said that the nine districts of the main city have supported the pattern of Chongqing as a big city. Therefore, if the main urban area is discussed separately from other districts and counties, the results may be different. Furthermore, future research can discuss all the districts and counties in Chongqing and conduct more detailed research on the coupling coordination relationship between the social economy and ecological environment in Chongqing.

## 6. Conclusions and Implications

This paper is based on the panel data of 16 cities in the Chengdu–Chongqing urban agglomeration from 2009 to 2018 using the CCD model to measure the CCD of the social economy and ecological environment under the concept of high-quality development. Through regression analysis, the factors influencing the CCD of these two systems are explored. The following points are concluded:

(1) The overall level of the CCD between the social economy and ecological environment of the Chengdu–Chongqing urban agglomeration is relatively low. Only Chongqing barely achieved basic coordination in 2018. Chengdu and Chongqing are firmly in the top two, followed by Deyang and Mianyang. In 2018, the coupling coordination degree in other cities improved to varying degrees. Although the process is relatively slow, there is an overall upward trend.
(2) Through regression analysis, the following factors influencing the coupling and coordination relationship between the social economy and ecological environment of the Chengdu–Chongqing urban agglomeration are explored: urbanization rate, ratio of actual use of foreign capital and GDP, ratio of total export-import volume and GDP, the proportion of days with good air quality, and per capita public green space area, which can provide certain theoretical basis and policy guidance for policymakers.

This paper provides further explanation of the theory of ecological economics and enriches the theory of urban planning. The Chengdu–Chongqing urban agglomeration is an important area for China’s rapid economic development and is also China’s fourth pole of economic growth. The coupling coordination relationship between the social economy and ecological environment of the Chengdu–Chongqing urban agglomeration explores the human activity influence on the ecological environment, which enriches ecological and regional economic theories as well as urban planning, particularly the research of urban agglomerations. It provides more powerful theoretical support and empirical cases.

According to the connotation of the “Chengdu–Chongqing Urban Agglomeration Development Plan” and “Planning Outline for the Construction of Double Cities Economic Circle in Chengdu–Chongqing Region,” to improve the CCD of the social economy and ecological environment of the Chengdu–Chongqing urban agglomeration and promote sustainable development, this paper makes the following three suggestions based on the research conclusions:

(1) Give support to the leading role of Chengdu and Chongqing to break the restriction of administrative divisions on the development of urban agglomerations. The government should strengthen macro-control, promote the coordinated development of regional economy rather than the development of administrative regions, expand the radiation and influence of the two big cities, and avoid the disease of big cities. The two central cities should strengthen cooperation and share resources with other cities. Emphasizing the connecting role of the middle city Ziyang is necessary. Only strengthening the high-quality development of the middle prefecture-level cities in the Chengdu–Chongqing region can result in the high-quality development of the whole Chengdu–Chongqing urban agglomeration, driving the economic circle.
(2) Persist with reform and opening up, vigorously introduce high-quality foreign capital, and optimize the economic structure. Governments should select foreign companies based on their merits, upgrade the selection and evaluation criteria for foreign companies, select more environmentally friendly companies, and strictly control high-pollution and high-energy consumption enterprises from entering the local area. Quality foreign investment can not only drive local economic growth, but its demonstration effect and spillover effects can also help China’s industrial structural reform, improving the efficiency of resource allocation and promoting sustainable development.
(3) Increase investment and supervision of environmental governance and vigorously build ecological cities. Governments must increase research and support for green technologies and establish a green development policy system [29], increase financial investment in regional environmental governance, develop new energy technologies, and build urban ecological parks. Simultaneously, considering the uneven development, governments should allocate resources reasonably, particularly for enterprises in industrial cities, such as Deyang and cities with more factories to increase supervision, and provide more support to provide a strong guarantee for economic development to achieve high-quality development.

## Figures and Tables

**Table 1:** Index system and weight calculation results.

System	Subsystems	Indicators	Unit	Weight	Type
Social economy	Innovation	Patent authorization quantity per 10,000 population	Unit	0.095	+
		Ratio of science and technology expenditure and the public finance expenditure	%	0.035	+
		R&D expenditure internal expenditure	%	0.183	+
	Coordination	Per capita GDP	10,000 Yuan/person	0.030	+
		Urbanization rate	%	0.020	+
		Productivity of social labor	Yuan/person	0.020	+
	Opening up	Ratio of actual use of foreign capital and GDP	%	0.118	+
		Ratio of total export-import volume and GDP	%	0.116	+
	Shareability	Ratio of education expenditure and GDP	%	0.040	+
		Number of college students	person	0.153	+
		Beds of hospitals per 10,000 population	Unit	0.006	+
Ecological environment	Environmental level	Ratio of green coverage of built-up areas	%	0.002	+
		Percentage of days with good air quality	%	0.106	+
		Per capita park and green area	m^2^	0.038	+
	Environmental pressure	Waste gas discharge per unit of GDP	t/10,000 Yuan	0.005	_
		Wastewater discharge per unit of GDP	t/10,000 Yuan	0.008	_
	Environmental protection	Harmless treatment rate of domestic waste	%	0.009	+
		Sewage treatment rate	%	0.010	+
		Ratio of industrial solid wastes comprehensively utilized	%	0.006	+

**Table 2:** The CCD results.

**City**	**Coupling Coordination Degree**									
	**2009**	**2010**	**2011**	**2012**	**2013**	**2014**	**2015**	**2016**	**2017**	**2018**
Chongqing	0.26	0.29	0.33	0.35	0.30	0.32	0.35	0.37	0.38	0.40
Chengdu	0.30	0.33	0.35	0.35	0.31	0.31	0.31	0.32	0.35	0.38
Zigong	0.14	0.15	0.15	0.17	0.14	0.13	0.14	0.13	0.13	0.15
Luzhou	0.12	0.12	0.14	0.15	0.14	0.13	0.14	0.13	0.16	0.19
Deyang	0.17	0.18	0.19	0.20	0.19	0.19	0.17	0.15	0.17	0.27
Mianyang	0.20	0.19	0.22	0.22	0.22	0.21	0.22	0.22	0.23	0.26
Suining	0.12	0.14	0.14	0.16	0.15	0.15	0.14	0.15	0.15	0.16
Neijiang	0.12	0.14	0.15	0.15	0.14	0.15	0.13	0.13	0.14	0.16
Leshan	0.15	0.15	0.15	0.15	0.15	0.17	0.14	0.14	0.14	0.17
Nanchong	0.14	0.14	0.14	0.16	0.14	0.14	0.13	0.13	0.15	0.15
Meishan	0.14	0.14	0.15	0.16	0.15	0.16	0.11	0.13	0.14	0.16
Yibin	0.13	0.14	0.15	0.16	0.15	0.15	0.15	0.14	0.14	0.15
Guang’an	0.10	0.12	0.13	0.14	0.15	0.14	0.13	0.13	0.14	0.15
Dazhou	0.10	0.10	0.11	0.12	0.13	0.14	0.12	0.12	0.16	0.14
Ya’an	0.12	0.13	0.14	0.17	0.15	0.16	0.16	0.15	0.15	0.17
Ziyang	0.12	0.13	0.14	0.14	0.14	0.16	0.13	0.12	0.14	0.14

**Table 3:** Variation of the CCD.

City	2009	Ranking	2012	Ranking	2015	Ranking	2018	Ranking	CCD Variation	Ranking Variation
Chengdu	0.30	1	0.35	1	0.31	2	0.38	2	0.08	−1
Chongqi-ng	0.26	2	0.35	1	0.35	1	0.40	1	0.14	+1
Mianyang	0.20	3	0.22	3	0.22	3	0.26	4	0.06	−1
Deyang	0.17	4	0.20	4	0.17	4	0.27	3	0.10	+1
Leshan	0.15	5	0.15	11	0.14	7	0.17	6	0.02	−1
Zigong	0.14	6	0.17	5	0.14	8	0.15	11	0.01	−5
Nanchong	0.14	6	0.16	7	0.13	11	0.15	12	0.01	−6
Meishan	0.14	6	0.16	8	0.11	16	0.16	8	0.02	−2
Yibin	0.13	9	0.16	9	0.15	6	0.15	13	0.02	−4
Luzhou	0.12	10	0.15	12	0.14	9	0.19	5	0.07	+5
Suining	0.12	10	0.16	10	0.14	10	0.16	9	0.04	+1
Neijiang	0.12	10	0.15	13	0.13	12	0.16	10	0.04	0
Ya’an	0.12	10	0.17	5	0.16	5	0.17	7	0.05	+3
Ziyang	0.12	10	0.14	14	0.13	13	0.14	15	0.02	−5
Guang’an	0.10	15	0.14	15	0.13	14	0.15	14	0.05	+1
Dazhou	0.10	16	0.12	16	0.12	15	0.14	16	0.04	0

**Table 4:** The classification of the CCD.

Coordination Degree	0.00 < D ≤ 0.20	0.21 < D ≤ 0.40	0.41 < D ≤ 0.60	0.61 < D ≤ 0.80	0.81 < D ≤ 1.00
Classification	No coordination	Low coordination	Basic coordination	Good coordination	Excellent coordination

**Table 5:** The global Moran’s I index from 2009 to 2018.

Year	2009	2012	2015	2018
The Global Moran index	−0.190	−0.201	−0.187	−0.110

**Table 6:** Regression analysis results.

	X1	X2	X3	X4	X5	X6	X7	X8	X9	X10	X11
CCD	0.03 ***	0.03 ***	0.04 ***	0.02 ***	0.06 ***	0.01	0.06 ***	0.05 ***	0.03 ***	−0.04	0.02 ***
	(0.01)	(0.01)	(0.01)	(0.01)	(0.02)	(0.01)	(0.01)	(0.01)	(0.01)	(0.02)	(0.01)
	X12	X13	X14	X15	X16	X17	X18	X19	_cons	N	R^2^
	0.00	0.07 ***	0.06 ***	0.00	0.01 **	−0.00	0.01 ***	0.00	0.03 ***	160	0.961
	(0.00)	(0.00)	(0.01)	(0.00)	(0.00)	(0.00)	(0.00)	(0.00)	(0.01)		

Standard errors in parentheses ** *p* < 0.05, *** *p* < 0.01.

## Data Availability

The raw/processed data required to reproduce these findings cannot be shared at this time as the data also forms part of an ongoing study.
